# 2-Amino-4-(2,4-dichloro­phen­yl)-6-(naphthalen-1-yl)nicotinonitrile

**DOI:** 10.1107/S1600536810050609

**Published:** 2010-12-11

**Authors:** Yuan-Feng Ye, Huai-Qing Wang, Zhi-Qiang Feng

**Affiliations:** a School of Material Engineering, Jinling Institute of Technology, Nanjing 211169, People’s Republic of China

## Abstract

In the crystal structure of the title compound, C_22_H_13_Cl_2_N_3_, the mol­ecules are connected *via* inter­molecular C—H⋯N and N—H⋯N hydrogen bonds, forming a three-dimensional network. The dihedral angles between naphthyl ring system and the pyridyl and benzene rings are 55.04 (7) and 75.87 (7)°, respectively, whereas the pyridyl and benzene rings are oriented at a dihedral angle of 59.56 (8)°.

## Related literature

For the synthetic procedure, see: Mantri *et al.* (2008 ▶). For the use of the title compound in the preparation of medicines, see: Mkhalid *et al.* (2006 ▶). For general background to this type of compound, see: Moreau & Huber (1999 ▶).
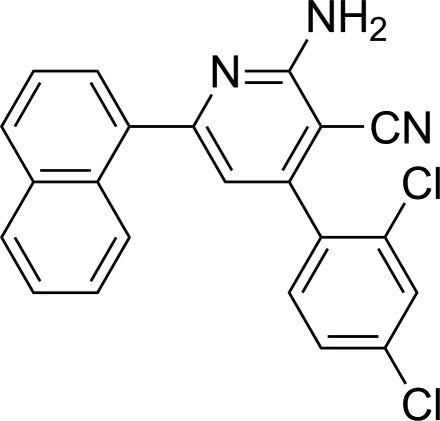


## Experimental

### 

#### Crystal data

C_22_H_13_Cl_2_N_3_
*M* *_r_* = 390.25
Triclinic, 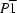

*a* = 9.5020 (19) Å
*b* = 10.054 (2) Å
*c* = 10.735 (2) Å
α = 72.78 (3)°
β = 89.17 (3)°
γ = 74.81 (3)°
*V* = 943.1 (3) Å^3^
*Z* = 2
Mo *K*α radiation
μ = 0.36 mm^−1^
*T* = 293 K
0.30 × 0.10 × 0.10 mm

#### Data collection

Entaf–Nonius CAD-4 diffractometer
Absorption correction: ψ scan (North *et al.*, 1968 ▶) *T*_min_ = 0.901, *T*_max_ = 0.965
3686 measured reflections
3463 independent reflections
2648 reflections with *I* > 2σ(*I*)
*R*_int_ = 0.014
3 standard reflections every 200 reflections  intensity decay: 1%

#### Refinement

*R*[*F* ^2^ > 2σ(*F* ^2^)] = 0.048
*wR*(*F* ^2^) = 0.146
*S* = 1.00
3463 reflections
252 parameters
H atoms treated by a mixture of independent and constrained refinement
Δρ_max_ = 0.18 e Å^−3^
Δρ_min_ = −0.37 e Å^−3^

### 

Data collection: *CAD-4 Software* (Enraf–Nonius, 1985 ▶); cell refinement: *CAD-4 Software*; data reduction: *XCAD4* (Harms & Wocadlo, 1995 ▶); program(s) used to solve structure: *SHELXS97* (Sheldrick, 2008 ▶); program(s) used to refine structure: *SHELXL97* (Sheldrick, 2008 ▶); molecular graphics: *SHELXTL* (Sheldrick, 2008 ▶); software used to prepare material for publication: *SHELXTL*.

## Supplementary Material

Crystal structure: contains datablocks I, global. DOI: 10.1107/S1600536810050609/bq2258sup1.cif

Structure factors: contains datablocks I. DOI: 10.1107/S1600536810050609/bq2258Isup2.hkl

Additional supplementary materials:  crystallographic information; 3D view; checkCIF report

## Figures and Tables

**Table 1:** Hydrogen-bond geometry (Å, °)

*D*—H⋯*A*	*D*—H	H⋯*A*	*D*⋯*A*	*D*—H⋯*A*
N2—H2*A*⋯N1^i^	0.85 (3)	2.19 (3)	3.034 (3)	176 (2)
C4—H4*A*⋯N3^ii^	0.93	2.62	3.488 (4)	155

Symmetry codes: (i) 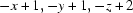; (ii) 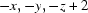.

**Fig. 1.**
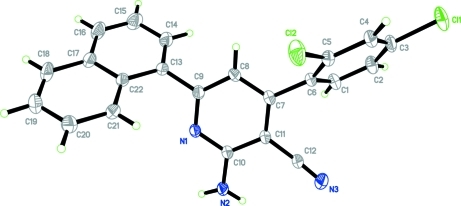
The molecular structure of (I), with the atom-numbering scheme. Displacement ellipsoids are drawn at the 50% probability level.

**Fig. 2.**
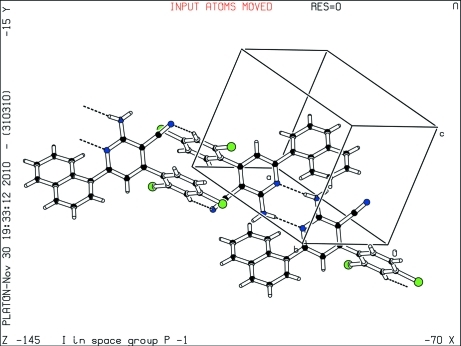
A packing diagram for (I). C—H···N and N—H···N hydrogen bonds are shown by dashed lines.

## supplementary crystallographic information

### Comment

The title compound, C_22_H_13_Cl_2_N_3_,(I), contains amino group, which can react with different groups to prepare various function organic compounds. It is a kind of aromatic organic intermediate which can be used for many fields such as medicine (Mantri *et al.*, 2008). The molecular structure of (I) is shown in Fig. 1. In (I), the naphthyl and the two rings, pyridyl and phenyl are oriented with different dihedral angles; 55.04 (7) ° between naphthyl and pyridyl, 75.87 (7) ° between naphthyl and phenyl and 59.56 (8) ° between pyridyl and phenyl. In the crystal structure of the title compound, the molecules were connected together *via* N—H···N and C—H···N intermolecular hydrogen bonds to form a three dimensional network, which seems to be very effective in the stabilization of the crystal structure.

### Experimental

The title compound, (I) was prepared by the literature method (Mantri *et al.*, 2008). Crystals suitable for X-ray analysis were obtained by dissolving (I) (0.5 g) in methanol (20 ml) and evaporating the solvent slowly at room temperature for about 5 d.

### Refinement

All H atoms were positioned geometrically and constrained to ride on their parent atoms, with C—H = 0.93 Å for aromatic H and 0.86 Å for N—H, respectively. The *U*_iso_(H) = *xU*_eq_(C), where *x* = 1.2 for aromatic H, and *x* = 1.5 for other H.

### Crystal data

**Table:**

C_22_H_13_Cl_2_N_3_	*Z* = 2
*M**_r_* = 390.25	*F*(000) = 400
Triclinic, *P*1	*D*_x_ = 1.374 Mg m^−^^3^
Hall symbol: -P 1	Mo *K*α radiation, λ = 0.71073 Å
*a* = 9.5020 (19) Å	Cell parameters from 25 reflections
*b* = 10.054 (2) Å	θ = 10–14°
*c* = 10.735 (2) Å	µ = 0.36 mm^−^^1^
α = 72.78 (3)°	*T* = 293 K
β = 89.17 (3)°	Block, colourless
γ = 74.81 (3)°	0.30 × 0.10 × 0.10 mm
*V* = 943.1 (3) Å^3^	

### Data collection

**Table:**

Entaf–Nonius CAD-4 diffractometer	2648 reflections with *I* > 2σ(*I*)
Radiation source: fine-focus sealed tube	*R*_int_ = 0.014
graphite	θ_max_ = 25.4°, θ_min_ = 2.0°
ω/2θ scans	*h* = 0→11
Absorption correction: ψ scan (North *et al.*, 1968)	*k* = −11→12
*T*_min_ = 0.901, *T*_max_ = 0.965	*l* = −12→12
3686 measured reflections	3 standard reflections every 200 reflections
3463 independent reflections	intensity decay: 1%

### Refinement

**Table:**

Refinement on *F*^2^	Primary atom site location: structure-invariant direct methods
Least-squares matrix: full	Secondary atom site location: difference Fourier map
*R*[*F*^2^ > 2σ(*F*^2^)] = 0.048	Hydrogen site location: inferred from neighbouring sites
*wR*(*F*^2^) = 0.146	H atoms treated by a mixture of independent and constrained refinement
*S* = 1.00	*w* = 1/[σ^2^(*F*_o_^2^) + (0.095*P*)^2^ + ] where *P* = (*F*_o_^2^ + 2*F*_c_^2^)/3
3463 reflections	(Δ/σ)_max_ < 0.001
252 parameters	Δρ_max_ = 0.18 e Å^−^^3^
0 restraints	Δρ_min_ = −0.37 e Å^−^^3^

### Special details

**Table:**

Geometry. All e.s.d.'s (except the e.s.d. in the dihedral angle between two l.s. planes) are estimated using the full covariance matrix. The cell e.s.d.'s are taken into account individually in the estimation of e.s.d.'s in distances, angles and torsion angles; correlations between e.s.d.'s in cell parameters are only used when they are defined by crystal symmetry. An approximate (isotropic) treatment of cell e.s.d.'s is used for estimating e.s.d.'s involving l.s. planes.
Refinement. Refinement of *F*^2^ against ALL reflections. The weighted *R*-factor *wR* and goodness of fit *S* are based on *F*^2^, conventional *R*-factors *R* are based on *F*, with *F* set to zero for negative *F*^2^. The threshold expression of *F*^2^ > σ(*F*^2^) is used only for calculating *R*-factors(gt) *etc*. and is not relevant to the choice of reflections for refinement. *R*-factors based on *F*^2^ are statistically about twice as large as those based on *F*, and *R*- factors based on ALL data will be even larger.

### Fractional atomic coordinates and isotropic or equivalent isotropic displacement parameters (Å^2^)

**Table:**

	*x*	*y*	*z*	*U*_iso_*/*U*_eq_	
Cl1	−0.37446 (8)	0.08316 (9)	0.92700 (9)	0.0687 (3)	
N1	0.36411 (19)	0.4640 (2)	0.89712 (17)	0.0331 (4)	
C1	−0.0992 (3)	0.2943 (3)	0.9925 (3)	0.0425 (6)	
H1B	−0.0942	0.3531	1.0439	0.051*	
Cl2	0.13996 (8)	0.12749 (9)	0.73801 (8)	0.0635 (3)	
N2	0.4248 (2)	0.3529 (3)	1.1161 (2)	0.0414 (5)	
H2A	0.480 (3)	0.408 (3)	1.110 (2)	0.044 (7)*	
H2B	0.398 (3)	0.313 (3)	1.193 (3)	0.053 (8)*	
C2	−0.2176 (3)	0.2375 (3)	0.9987 (3)	0.0477 (6)	
H2C	−0.2919	0.2580	1.0532	0.057*	
C3	−0.2235 (3)	0.1508 (3)	0.9234 (3)	0.0442 (6)	
N3	0.1702 (3)	0.1711 (3)	1.2494 (2)	0.0542 (6)	
C4	−0.1143 (3)	0.1165 (3)	0.8436 (2)	0.0426 (6)	
H4A	−0.1192	0.0557	0.7942	0.051*	
C5	0.0034 (3)	0.1752 (3)	0.8384 (2)	0.0390 (6)	
C6	0.0130 (2)	0.2659 (2)	0.9114 (2)	0.0350 (5)	
C7	0.1356 (2)	0.3340 (2)	0.9053 (2)	0.0347 (5)	
C8	0.1657 (3)	0.4256 (3)	0.7898 (2)	0.0394 (6)	
H8A	0.1114	0.4431	0.7125	0.047*	
C9	0.2771 (2)	0.4911 (2)	0.7898 (2)	0.0353 (5)	
C10	0.3364 (2)	0.3766 (2)	1.0103 (2)	0.0315 (5)	
C11	0.2194 (2)	0.3119 (2)	1.0184 (2)	0.0327 (5)	
C12	0.1897 (2)	0.2309 (3)	1.1454 (2)	0.0372 (5)	
C13	0.3005 (2)	0.6054 (3)	0.6730 (2)	0.0364 (5)	
C14	0.1836 (3)	0.7200 (3)	0.6165 (3)	0.0528 (7)	
H14A	0.0907	0.7181	0.6441	0.063*	
C15	0.2017 (4)	0.8417 (3)	0.5168 (3)	0.0660 (9)	
H15A	0.1209	0.9184	0.4783	0.079*	
C16	0.3370 (3)	0.8461 (3)	0.4774 (3)	0.0615 (8)	
H16A	0.3486	0.9278	0.4140	0.074*	
C17	0.4602 (3)	0.7293 (3)	0.5308 (2)	0.0459 (6)	
C18	0.6025 (3)	0.7317 (4)	0.4896 (3)	0.0615 (8)	
H18A	0.6156	0.8140	0.4280	0.074*	
C19	0.7199 (3)	0.6162 (4)	0.5385 (3)	0.0623 (8)	
H19A	0.8128	0.6208	0.5126	0.075*	
C20	0.7004 (3)	0.4907 (3)	0.6275 (3)	0.0520 (7)	
H20A	0.7801	0.4101	0.6576	0.062*	
C21	0.5663 (3)	0.4846 (3)	0.6708 (2)	0.0411 (6)	
H21A	0.5561	0.3999	0.7306	0.049*	
C22	0.4431 (3)	0.6037 (3)	0.6270 (2)	0.0355 (5)	

### Atomic displacement parameters (Å^2^)

**Table:**

	*U*^11^	*U*^22^	*U*^33^	*U*^12^	*U*^13^	*U*^23^
Cl1	0.0450 (4)	0.0707 (5)	0.1083 (7)	−0.0386 (4)	0.0167 (4)	−0.0338 (5)
N1	0.0318 (10)	0.0407 (11)	0.0316 (10)	−0.0177 (8)	0.0053 (8)	−0.0112 (8)
C1	0.0358 (13)	0.0445 (14)	0.0554 (15)	−0.0182 (11)	0.0083 (11)	−0.0211 (12)
Cl2	0.0653 (5)	0.0837 (5)	0.0692 (5)	−0.0447 (4)	0.0347 (4)	−0.0439 (4)
N2	0.0432 (12)	0.0565 (14)	0.0308 (11)	−0.0301 (11)	0.0007 (9)	−0.0078 (10)
C2	0.0350 (13)	0.0484 (15)	0.0667 (17)	−0.0175 (11)	0.0154 (12)	−0.0227 (13)
C3	0.0327 (13)	0.0409 (14)	0.0607 (16)	−0.0206 (11)	0.0027 (11)	−0.0083 (12)
N3	0.0611 (15)	0.0587 (14)	0.0428 (13)	−0.0297 (12)	0.0063 (11)	−0.0038 (11)
C4	0.0444 (14)	0.0422 (14)	0.0486 (14)	−0.0229 (12)	0.0031 (11)	−0.0148 (11)
C5	0.0370 (13)	0.0454 (14)	0.0371 (12)	−0.0202 (11)	0.0064 (10)	−0.0083 (11)
C6	0.0315 (12)	0.0369 (12)	0.0376 (12)	−0.0161 (10)	−0.0002 (10)	−0.0068 (10)
C7	0.0308 (11)	0.0372 (12)	0.0412 (13)	−0.0154 (10)	0.0055 (10)	−0.0141 (10)
C8	0.0367 (13)	0.0532 (15)	0.0345 (12)	−0.0242 (11)	0.0004 (10)	−0.0117 (11)
C9	0.0351 (12)	0.0412 (13)	0.0338 (12)	−0.0166 (10)	0.0067 (10)	−0.0124 (10)
C10	0.0307 (11)	0.0338 (12)	0.0339 (12)	−0.0131 (9)	0.0056 (9)	−0.0122 (9)
C11	0.0314 (11)	0.0344 (12)	0.0359 (12)	−0.0149 (9)	0.0062 (9)	−0.0110 (10)
C12	0.0361 (12)	0.0378 (13)	0.0418 (14)	−0.0186 (10)	0.0031 (10)	−0.0106 (11)
C13	0.0376 (13)	0.0434 (13)	0.0310 (12)	−0.0179 (11)	0.0030 (10)	−0.0092 (10)
C14	0.0411 (15)	0.0617 (18)	0.0483 (15)	−0.0126 (13)	0.0071 (12)	−0.0071 (13)
C15	0.0593 (19)	0.0566 (18)	0.0579 (18)	−0.0013 (15)	0.0045 (15)	0.0059 (14)
C16	0.0656 (19)	0.0498 (17)	0.0534 (17)	−0.0141 (15)	0.0135 (14)	0.0057 (13)
C17	0.0513 (15)	0.0503 (15)	0.0363 (13)	−0.0222 (13)	0.0078 (11)	−0.0062 (11)
C18	0.065 (2)	0.069 (2)	0.0523 (16)	−0.0370 (17)	0.0211 (14)	−0.0053 (15)
C19	0.0465 (16)	0.088 (2)	0.0569 (18)	−0.0307 (17)	0.0174 (14)	−0.0192 (16)
C20	0.0407 (14)	0.0675 (18)	0.0458 (15)	−0.0114 (13)	0.0085 (12)	−0.0174 (14)
C21	0.0445 (14)	0.0472 (14)	0.0315 (12)	−0.0153 (12)	0.0056 (10)	−0.0094 (11)
C22	0.0396 (13)	0.0437 (13)	0.0290 (11)	−0.0191 (11)	0.0039 (9)	−0.0125 (10)

### Geometric parameters (Å, °)

**Table:**

Cl1—C3	1.736 (2)	C9—C13	1.488 (3)
N1—C10	1.341 (3)	C10—C11	1.417 (3)
N1—C9	1.345 (3)	C11—C12	1.434 (3)
C1—C2	1.382 (3)	C13—C14	1.365 (4)
C1—C6	1.392 (3)	C13—C22	1.432 (3)
C1—H1B	0.9300	C14—C15	1.414 (4)
Cl2—C5	1.736 (2)	C14—H14A	0.9300
N2—C10	1.347 (3)	C15—C16	1.356 (4)
N2—H2A	0.84 (3)	C15—H15A	0.9300
N2—H2B	0.86 (3)	C16—C17	1.406 (4)
C2—C3	1.365 (4)	C16—H16A	0.9300
C2—H2C	0.9300	C17—C18	1.421 (4)
C3—C4	1.376 (3)	C17—C22	1.421 (3)
N3—C12	1.139 (3)	C18—C19	1.358 (4)
C4—C5	1.388 (3)	C18—H18A	0.9300
C4—H4A	0.9300	C19—C20	1.394 (4)
C5—C6	1.387 (3)	C19—H19A	0.9300
C6—C7	1.490 (3)	C20—C21	1.360 (4)
C7—C8	1.386 (3)	C20—H20A	0.9300
C7—C11	1.390 (3)	C21—C22	1.405 (3)
C8—C9	1.384 (3)	C21—H21A	0.9300
C8—H8A	0.9300		
			
C10—N1—C9	118.57 (19)	C7—C11—C10	119.3 (2)
C2—C1—C6	121.8 (2)	C7—C11—C12	123.0 (2)
C2—C1—H1B	119.1	C10—C11—C12	117.6 (2)
C6—C1—H1B	119.1	N3—C12—C11	175.9 (3)
C10—N2—H2A	117.4 (17)	C14—C13—C22	120.1 (2)
C10—N2—H2B	118.6 (19)	C14—C13—C9	118.0 (2)
H2A—N2—H2B	118 (2)	C22—C13—C9	121.7 (2)
C3—C2—C1	118.7 (2)	C13—C14—C15	121.0 (3)
C3—C2—H2C	120.7	C13—C14—H14A	119.5
C1—C2—H2C	120.7	C15—C14—H14A	119.5
C2—C3—C4	122.0 (2)	C16—C15—C14	119.8 (3)
C2—C3—Cl1	119.0 (2)	C16—C15—H15A	120.1
C4—C3—Cl1	118.96 (19)	C14—C15—H15A	120.1
C3—C4—C5	118.3 (2)	C15—C16—C17	121.2 (3)
C3—C4—H4A	120.8	C15—C16—H16A	119.4
C5—C4—H4A	120.8	C17—C16—H16A	119.4
C6—C5—C4	121.7 (2)	C16—C17—C18	121.9 (3)
C6—C5—Cl2	121.38 (17)	C16—C17—C22	119.7 (2)
C4—C5—Cl2	116.88 (19)	C18—C17—C22	118.4 (2)
C5—C6—C1	117.4 (2)	C19—C18—C17	121.3 (3)
C5—C6—C7	123.1 (2)	C19—C18—H18A	119.3
C1—C6—C7	119.5 (2)	C17—C18—H18A	119.3
C8—C7—C11	118.0 (2)	C18—C19—C20	119.6 (3)
C8—C7—C6	121.8 (2)	C18—C19—H19A	120.2
C11—C7—C6	120.2 (2)	C20—C19—H19A	120.2
C9—C8—C7	119.8 (2)	C21—C20—C19	120.8 (3)
C9—C8—H8A	120.1	C21—C20—H20A	119.6
C7—C8—H8A	120.1	C19—C20—H20A	119.6
N1—C9—C8	122.7 (2)	C20—C21—C22	121.4 (2)
N1—C9—C13	115.38 (19)	C20—C21—H21A	119.3
C8—C9—C13	121.8 (2)	C22—C21—H21A	119.3
N1—C10—N2	117.0 (2)	C21—C22—C17	118.2 (2)
N1—C10—C11	121.53 (19)	C21—C22—C13	123.8 (2)
N2—C10—C11	121.4 (2)	C17—C22—C13	118.0 (2)
			
C6—C1—C2—C3	0.3 (4)	N2—C10—C11—C7	−176.9 (2)
C1—C2—C3—C4	1.1 (4)	N1—C10—C11—C12	−174.4 (2)
C1—C2—C3—Cl1	−178.1 (2)	N2—C10—C11—C12	5.6 (3)
C2—C3—C4—C5	−1.3 (4)	C7—C11—C12—N3	−155 (4)
Cl1—C3—C4—C5	177.91 (18)	C10—C11—C12—N3	22 (4)
C3—C4—C5—C6	0.1 (4)	N1—C9—C13—C14	−123.8 (2)
C3—C4—C5—Cl2	179.22 (19)	C8—C9—C13—C14	51.5 (3)
C4—C5—C6—C1	1.2 (4)	N1—C9—C13—C22	50.6 (3)
Cl2—C5—C6—C1	−177.86 (18)	C8—C9—C13—C22	−134.1 (2)
C4—C5—C6—C7	−178.2 (2)	C22—C13—C14—C15	−3.2 (4)
Cl2—C5—C6—C7	2.7 (3)	C9—C13—C14—C15	171.4 (3)
C2—C1—C6—C5	−1.4 (4)	C13—C14—C15—C16	−0.9 (5)
C2—C1—C6—C7	178.0 (2)	C14—C15—C16—C17	2.3 (5)
C5—C6—C7—C8	59.9 (3)	C15—C16—C17—C18	179.4 (3)
C1—C6—C7—C8	−119.5 (3)	C15—C16—C17—C22	0.4 (5)
C5—C6—C7—C11	−123.1 (3)	C16—C17—C18—C19	−177.6 (3)
C1—C6—C7—C11	57.5 (3)	C22—C17—C18—C19	1.3 (4)
C11—C7—C8—C9	0.3 (4)	C17—C18—C19—C20	2.2 (5)
C6—C7—C8—C9	177.4 (2)	C18—C19—C20—C21	−3.1 (4)
C10—N1—C9—C8	−4.0 (3)	C19—C20—C21—C22	0.4 (4)
C10—N1—C9—C13	171.27 (19)	C20—C21—C22—C17	3.1 (4)
C7—C8—C9—N1	3.5 (4)	C20—C21—C22—C13	−177.5 (2)
C7—C8—C9—C13	−171.4 (2)	C16—C17—C22—C21	175.1 (2)
C9—N1—C10—N2	−179.4 (2)	C18—C17—C22—C21	−3.9 (4)
C9—N1—C10—C11	0.7 (3)	C16—C17—C22—C13	−4.4 (4)
C8—C7—C11—C10	−3.4 (3)	C18—C17—C22—C13	176.7 (2)
C6—C7—C11—C10	179.4 (2)	C14—C13—C22—C21	−173.7 (2)
C8—C7—C11—C12	173.9 (2)	C9—C13—C22—C21	12.0 (3)
C6—C7—C11—C12	−3.3 (3)	C14—C13—C22—C17	5.7 (3)
N1—C10—C11—C7	3.1 (3)	C9—C13—C22—C17	−168.6 (2)

### Hydrogen-bond geometry (Å, °)

**Table:**

*D*—H···*A*	*D*—H	H···*A*	*D*···*A*	*D*—H···*A*
N2—H2A···N1^i^	0.85 (3)	2.19 (3)	3.034 (3)	176 (2)
C4—H4A···N3^ii^	0.93	2.62	3.488 (4)	155

Symmetry codes: (i) −*x*+1, −*y*+1, −*z*+2; (ii) −*x*, −*y*, −*z*+2.

## References

[bb1] Enraf–Nonius (1985). *CAD-4 Software* Enraf–Nonius, Delft, The Netherlands.

[bb2] Harms, K. & Wocadlo, S. (1995). *XCAD4* University of Marburg, Germany.

[bb3] Mantri, M., Graaf, O., Veldhoven, J. & IJzerman, A. P. (2008). *J. Med. Chem.* **51**, 4449–4455.

[bb4] Mkhalid, I. A. I., Coventry, D. N., Albesa-Jove, D., Batsanov, A. S., Howard, J. A. K. & Marder, T. B. (2006). *Angew. Chem. Int. Ed.* **45**, 489–491. 10.1002/anie.200503047 16323236

[bb5] Moreau, J. L. & Huber, G. (1999). *Brain. Res. Rev.* **31**, 65–82. 10.1016/s0165-0173(99)00059-4 10611496

[bb6] North, A. C. T., Phillips, D. C. & Mathews, F. S. (1968). *Acta Cryst.* A**24**, 351–359.

[bb7] Sheldrick, G. M. (2008). *Acta Cryst.* A**64**, 112–122. 10.1107/S0108767307043930 18156677

